# A high IL6/IL17 ratio combined with low IL5 expression is correlated with poor survival in squamous cervical cancer

**DOI:** 10.1186/2051-1426-2-S3-P232

**Published:** 2014-11-06

**Authors:** Simone Punt, Jeanine J Houwing, Iris A Schulkens, Victor L Thijssen, Elisabeth M Osse, Eva Kritikou, Cornelis D de Kroon, Arjan W Griffioen, Gert Jan Fleuren, Arko Gorter, Ekaterina S Jordanova

**Affiliations:** 1 Leiden University Medical Center, Leiden, Netherlands; 2 VU University medical center, Amsterdam, Netherlands; 3 Leiden University, Leiden, Netherlands

## 

Cervical cancer is the second leading cause of cancer death in young women worldwide. The aim of the current study was to identify which combination of immunological and vascular factors in the tumor microenvironment of cervical carcinoma is most significant for survival. Using qRT-PCR, 42 markers were investigated in frozen squamous cervical cancer samples (n = 52). Weighted gene co-expression network analysis and mixed-model analyses were performed to identify gene expression clusters and study their correlations. Selected individual factors were further investigated by immunohistochemistry. We identified a 'T cells' (CD3E/CD8A/TBX21/IFNG/FOXP3/IDO1), 'Macrophages' (CD4/CD14/CD163), 'Th2' (IL4/IL5/IL13/IL12), 'Inflammation' (IL6/IL1B/IL8/IL23/IL10/ARG1), 'Angiogenesis' (VEGFA/FLT1/ANGPT2/ PGF/ICAM1) and 'Vessel maturation' (PECAM1/VCAM1/ANGPT1/SELE/KDR/LGALS9) cluster. The 'T cells' cluster significantly correlated with early TNM stage (p = 0.007). High expression of 'T cells' marker CD3E correlated with improved disease-specific survival (p = 0.022). High expression of 'Angiogenesis' marker VEGFA correlated with poor survival (p = 0.032). High expression of 'Inflammation' marker IL6 was an independent predictor of poor survival (hazard ratio = 2.3, p = 0.011). A high IL6/IL17 ratio combined with low IL5 expression gave a hazard ratio of 4.2 (p = 0.010). Using immunohistochemistry, we have shown that IL-6 is correlated with poor survival, and determined that IL-17 was predominantly expressed by neutrophils (66%), mast cells (23%) and innate lymphoid cells (8%). Remarkably, Th17 cells were only a minor IL-17 expressing population (4%). A similar distribution was found in head and neck, ovarian, endometrial, prostate, breast, lung and colon carcinoma. A high number of IL-17 expressing cells was an independent prognostic factor for poor survival in early stage disease (p = 0.016, n = 160). A high number of Th17 cells was an independent prognostic factor for improved disease-specific survival (p = 0.026), suggesting Th17 cells are part of an anti-tumor immune response. IL6 independently predicted poor survival in cervical cancer. IL17 expressed by Th17 cells could counteract the tumor promoting effects of IL6, even more so combined with a Th2 response characterized by IL5. Since RNA levels, in contrast to protein levels, are extremely low in activated neutrophils, the IL17 RNA levels measured most likely represented Th17 cells. Th17 cells, despite being a minor IL-17 expressing subpopulation, were also associated with improved survival in squamous cervical cancer. Total IL-17 expressing cells, primarily representing neutrophils, rather associated with poor survival in early stage disease. Measuring IL6, especially in combination with IL17 and IL5 expression, may improve the accuracy of predicting prognosis and support the development of anti-IL-6 combined with anti-VEGF-A therapy in cervical cancer.

